# Application of volumetric modulated arc therapy (VMAT) in a dual-vendor environment

**DOI:** 10.1186/1748-717X-5-95

**Published:** 2010-10-25

**Authors:** Barbara Dobler, Karin Weidner, Oliver Koelbl

**Affiliations:** 1 Department of Radiotherapy, Regensburg University Medical Center, D-93042 Regensburg, Germany

## Abstract

**Background and Purpose:**

The purpose of this study was to assess plan quality and treatment time achievable with the new VMAT optimization tool implemented in the treatment planning system Oncentra MasterPlan^® ^as compared to IMRT for Elekta SynergyS^® ^linear accelerators.

**Materials and methods:**

VMAT was implemented on a SynergyS^® ^linear accelerator (Elekta Ltd., Crawley, UK) with Mosaiq^® ^record and verify system (IMPAC Medical Systems, Sunnyvale, CA) and the treatment planning system Oncentra MasterPlan^® ^(Nucletron BV, Veenendaal, the Netherlands). VMAT planning was conducted for three typical target types of prostate cancer, hypopharynx/larynx cancer and vertebral metastases, and compared to standard IMRT with respect to plan quality, number of monitor units (MU), and treatment time.

**Results:**

For prostate cancer and vertebral metastases single arc VMAT led to similar plan quality as compared to IMRT. For treatment of the hypopharynx/larynx cancer, a second arc was necessary to achieve sufficient plan quality. Treatment time was reduced in all cases to 35% to 43% as compared to IMRT. Times required for optimization and dose calculation, however, increased by a factor of 5.0 to 6.8.

**Conclusion:**

Similar or improved plan quality can be achieved with VMAT as compared to IMRT at reduced treatment times but increased calculation times.

## Background

Volumetric modulated arc therapy (VMAT) allows irradiation with simultaneously varying dose rate, gantry speed, collimator, and leaf positions. It has been first introduced by Otto in 2008 [1] and implemented for Varian linear accelerators as RapidArc^® ^[2-8]. Various treatment planning studies have been published, comparing RapidArc^® ^and dynamic intensity modulated radiation therapy (IMRT) or conventional stereotactic treatments with regard to plan quality, delivery time, and monitor units required per fraction dose [2,3,7,9-19], using either in-house developed treatment planning systems (TPS) or the Varian TPS Eclipse. For Elekta linear accelerators volumetric modulated arc therapy became available under the label VMAT in 2008. The only commercially available treatment planning system was ERGO++ (3D Line Medical Systems/Elekta Ltd, Crawley, UK), which, however, requires initial definition of sub-arcs and manual adaptation of the multileaf collimator (MLC) before automatic weight optimization and can therefore not be considered as a fully inverse planning system [20-23]. Fully inverse treatment planning systems for Elekta linear accelerators have become commercially available only recently. A few plan comparison studies have been published [24-26] using the treatment planning system Pinnacle (Philips Healthcare, Andover, MA). All of these studies showed similar plan quality at substantially reduced treatment times for VMAT as compared to IMRT. In December 2009 a new VMAT optimization tool, implemented in Oncentra MasterPlan^® ^v3.3, was released clinically, which allows VMAT optimization for Varian and Elekta linear accelerators with a linac-vendor independent planning system.

The aim of this study was to investigate the feasibility of VMAT with the new commercial combination of Oncentra MasterPlan^® ^(Nucletron BV, Veenendaal, the Netherlands) and SynergyS^® ^linear accelerators (Elekta Ltd, Crawley, United Kingdom). VMAT optimization was performed for typical target types usually treated with IMRT at our department and compared to standard IMRT with regard to plan quality, number of monitor units, and treatment time. Patients were selected for whom treatment times required for IMRT are critical due to possible intra-fractional organ movement or patient discomfort and who therefore might benefit substantially from the advancement from IMRT to VMAT.

## Methods

### Linear accelerator and record and verify system

A SynergyS^® ^linear accelerator with 6MV photons, equipped with a BeamModulator™ head, an iViewGT™ electronic portal imaging device, and an on-board cone-beam CT XVI is used for VMAT delivery. The BeamModulator™ head has a multileaf collimator which consists of 40 leaf pairs of 4 mm width at isocenter and allows unrestricted leaf interdigitation. Fixed diaphragms limit the maximum field size of 21 cm × 16 cm, and there are no moveable jaws. Minimum and maximum number of MU per degree of gantry rotation are 0.10 MU/° and 20.0 MU/° respectively, minimum MU per cm leaf travel is 0.30 MU/cm, maximum gantry speed is 6.00 °/s. Maximum leaf speed is 2.4 cm/s, the dynamic minimum leaf gap 0.14 cm, and the static minimum leaf gap 0.0 cm. The maximum nominal dose rate is 500 MU/min. Seven fixed dose rate levels are available, each half the dose rate of the next higher level, continuous variation is not possible. Actual dose rates may differ from nominal dose rates by ±25%. During VMAT delivery the fastest combination of dose rate, gantry speed and leaf speed is automatically selected by the linac control system Precise Desktop^® ^7.

### Treatment planning system

Treatment planning is performed with Oncentra MasterPlan^® ^v3.3 SP1, released clinically in December 2009, on a 64 bit Windows system with 8 GB RAM and 8-core processor. For beam data modeling the above mentioned VMAT specific parameters of the linac have to be defined. Since the TPS only allows for 5 different dose rates, two dose rates had to be omitted. We kept the 5 higher and omitted the two lower dose rates, because according to the literature the main advantage of VMAT as compared to IMRT is the short treatment time, which would be prolonged if higher dose rates would be omitted. This is also in concordance with Bedford's recommendation not to use dose rates below 75 MU/min to a large extent due to instabilities of the linac below 37 MU/min [27]. Since the linac automatically selects the fastest combination of gantry speed, leaf speed and dose rate, these parameters are only used to ensure compliance with machine constraints and estimate treatment time in the optimization. They are, however, not transferred to the linac and have therefore no influence on the delivery.

For treatment planning, beams are set up in the Beam Modeling module (BM), in which the treatment unit, energy and collimator angle are defined by the user. Gantry speed, leaf positions and dose rate are subject to optimization, the collimator angle, however, is kept constant at the predefined value for each arc. Other user defined parameters for the optimization include start gantry angle, rotation direction, arc length, gantry angle spacing between subsequent control points (2° to 6°), maximum delivery time, number of arcs, and constrained leaf motion in cm/°.

Optimization is performed in the Optimization Module, which allows the user to choose between the IMRT options "Intensity Modulation" (IM) with subsequent leaf sequencing, and the direct machine parameter optimization "Direct Step and Shoot" (DSS), and VMAT.

In DSS a fluence optimization with subsequent leaf sequencing is performed for static fields for a few iterations to get an initial guess for the segments. In the next step, the gradients of the objective function are calculated with respect to leaf positions and weights, allowing direct optimization of deliverable MLC segments, which leads to improved results as compared to IM [28-30].

VMAT optimization starts with a fluence optimization for gantry angle spacing of 24° and subsequent MLC sequencing, generating 2 segments per gantry angle. The segments are then spread out evenly and cloned to achieve the required gantry angle spacing as defined by the user. Based on this starting point a direct machine parameter optimization is performed, taking machine restrictions into account, followed by a final accurate dose calculation and segment weight optimization. The method is a successor of and very similar to the method described in [31], where new segments are created by linear interpolation instead of cloning.

Continuous delivery is discretized and approximated by the calculation of static beams separated by 2° to 6°, depending on the user defined gantry angle spacing. The result of the accurate dose calculation is used as starting point for an automatic second optimization run to improve results [32]. For more than one arc, the dual arc option is available, which groups the segments, such that the required leaf movement is reduced, i.e. one arc contains segments with leaves positioned more to the left, and the other more to the right. Detailed information about the optimizer has been published in [31].

Commissioning of the system combination of Oncentra MasterPlan^®^, Mosaiq^® ^and SynergyS^® ^for VMAT has been successfully completed with individual plan verifications within 3% dose tolerance and 3 mm distance to agreement. Validation has been performed by absolute 2D-dosimetry using the 2D-array MatriXX^*Evolution*^^® ^(IBA Dosimetry, Schwarzenbruck, Germany). A description of the commissioning procedure and detailed results, however, is beyond the scope of this study and will be published separately.

### Treatment planning feasibility study

For a selection of patients who had undergone or were currently under IMRT treatment at our department, VMAT plans were optimized and compared to the IMRT plans to assess plan quality achievable with VMAT. The feasibility study was performed on three patients with typical target geometries of head and neck and prostate cancer, as well as spinal cord sparing irradiation of vertebrae.

1. A 64 year old patient with prostate cancer, pT3b, pN0, cM0, R1, with a planning target volume (PTV) of 424.1 cm^3 ^and a boost volume of 241.7 cm^3^. The PTV covered the prostatic fossa and the region of seminal vesicles defined by pelvic CT with 8 mm margin for setup, organ motion and delineation uncertainties. Dose prescription was 60 Gy in 2 Gy fractions to the average of the PTV, and 70 Gy in 2 Gy fractions to the boost volume. The bladder, rectum and the femoral heads were delineated as organs at risk (OAR). The volumes of rectum and bladder, which were not overlapping with the PTV that was extended by an additional 0.8 cm margin, were used as help structures for optimization and evaluation of plan quality, referred to as "rectum - PTV" and "bladder - PTV" respectively. The feasibility study was performed for the first series only. Dose volume objectives (DVO) based on dose prescription and OAR tolerance doses are listed in table 1.

**Table 1:** Treatment plan comparison for prostate cancer

Structure	Parameter	DVO	Single arc	IMRT
**PTV**	D_50%_	Uniform	60.0 Gy	59.9 Gy
		
	H	Dose	5.5	7.0
		
	V_95%_	60 Gy	99.9%	97.2%

**Normal Tissue**	D_1%_	≤ 60.0 Gy	60.0 Gy	59.9 Gy

	D_10%_	≤ 30.0 Gy	29.9 Gy	31.7 Gy

	D_25%_	≤ 15.0 Gy	17.5 Gy	17.7 Gy

**Rectum**	D_1%_	-	60.7 Gy	60.2 Gy

**Rectum - PTV**	D_1%_	≤ 40.0 Gy	37.8 Gy	38.5 Gy

**Bladder**	D_1%_	-	61.5 Gy	62.2 Gy

**Bladder - PTV**	D_1%_	≤ 30.0 Gy	47.4 Gy	47.4 Gy

**Left Femoral Head**	D_50%_	-	28.5 Gy	29.9 Gy

**Right Femoral Head**	D_50%_	-	29.8 Gy	28.9 Gy

**Monitor Units**	MU/2.0 Gy	-	695	687

**Time**	Calculation	-	16:30 min	2:52 min

	Delivery	-	4:45 min	11:00 min

D_X% _is the dose delivered to X% of the volume in Gy, V_95% _the volume receiving 95% of the prescription dose in%, Homogeneity H = (D_5% _- D_95%_)/D_average_.

2. A 52 year old male patient with cancer of the hypopharynx/larynx T4, N2c, M0, with 626.2 cm^3 ^PTV, and 452.2 cm^3 ^boost volume. The definition of PTV and organs at risk was according to literature [33]. Dose prescription was 60 Gy in 2 Gy fractions to the average of the PTV, and 70 Gy in 2 Gy fractions to the average of the boost volume. The spinal cord, the brain stem, the parotids, the temporomandibular joint, the lung, and the lenses were delineated as OAR. The feasibility study was performed for the PTV only. Dose volume objectives based on dose prescription and OAR tolerance doses are listed in table 2.

**Table 2:** Treatment plan comparison cancer for hypopharynx/larynx

Structure	Parameter	DVO	Dual Arc	Single Arc	IMRT
**PTV**	D_50%_	Uniform	60.0 Gy	60.5 Gy	59.7 Gy
		
	H	Dose	7.0	9.4	8.0
		
	V_95%_	60 Gy	97.8%	95.4%	95.7%

**Normal Tissue**	D_1%_	≤ 60 Gy	58.4 Gy	58.1 Gy	57.8 Gy

	D_20%_	≤ 21 Gy	20.1 Gy	20.4 Gy	21.5 Gy

	D_60%_	≤ 4 Gy	1.9 Gy	1.9 Gy	2.0 Gy

**Left Parotid**	D_50%_	≤ 26 Gy	23.7 Gy	23.1 Gy	29.4 Gy

**Right Parotid**	D_50%_	≤ 26 Gy	20.6 Gy	23.3 Gy	26.4 Gy

**Spinal Cord**	D_1 ccm_	≤ 39 Gy	36.9 Gy	39.8 Gy	37.6 Gy

**Brain Stem**	D_1 ccm_	≤ 43 Gy	34.4 Gy	41.5 Gy	36.9 Gy

Left Joint*	D_50%_	-	2.6 Gy	2.7 Gy	2.9 Gy

Right Joint*	D_50%_	-	2.3 Gy	2.2 Gy	2.5 Gy

**Monitor Units**	MU/2.0 Gy	-	715	552	799

**Time**	Calculation	-	33:10 min	16:30 min	4:52 min

	Delivery	-	5:00 min	2:08 min	14:15 min

*temporomandibular joint

D_X% _and D_1 ccm _are the doses delivered to X% of the volume and 1 cm^3 ^respectively, V_95% _the volume receiving 95% of the prescription dose in%, Homogeneity H = (D_5% _- D_95%_)/D_average_.

3. A 70 year old female patient with metastases in the lumbar vertebra, with a volume of 342.8 cm^3 ^of the PTV and 60.7 cm^3 ^of the GTV. The PTV was defined as the whole vertebral body with a 5 mm margin, the definition of GTV based on tumour mass identified by nuclear magnetic resonance tomography. Dose prescription was 44 Gy to the average of the PTV in fractions of 2.0 Gy and 55 Gy to the average of the GTV volume in fractions of 2.5 Gy, treated as simultaneous integrated boost (SIB). The spinal cord and the kidneys were delineated as OAR. Dose volume objectives based on dose prescription and OAR tolerance doses are listed in table 3.

**Table 3:** Treatment plan comparison for metastases of the lumbar vertebra (SIB)

Structure	Parameter	DVO	Single Arc	IMRT
**GTV**	D_50%_	Uniform	55.0 Gy	55.0 Gy
		
	H	Dose	7.3	7.1
		
**GTV**	V_95%_	55 Gy	95.6%	95.9%

**PTV**	D_95%_	≥ 40.0 Gy	41.4 Gy	40.5 Gy

**Normal Tissue**	D_1%_	≤ 40.0 Gy	41.3 Gy	42.6 Gy

	D_20%_	≤ 15.0 Gy	13.6 Gy	8.0 Gy

	D_60%_	≤ 5.0 Gy	1.8 Gy	1.3 Gy

**Left Kidney**	D_40%_	≤ 10.0 Gy	9.4 Gy	7.8 Gy

**Right Kidney**	D_40%_	≤ 10.0 Gy	9.0 Gy	5.3 Gy

**Spinal Cord**	D_1 ccm_	≤ 45.0 Gy	41.7 Gy	41.1 Gy

**Monitor Units**	MU/2.5 Gy	-	698	736

**Time**	Calculation	-	13:50 min	2:45 min

	Delivery	-	4:30 min	11:00 min

D_X% _and D_1 ccm _are the doses delivered to X% of the volume and 1 cm^3 ^respectively, V_95% _the volume receiving 95% of the prescription dose in%, Homogeneity H = (D_5% _- D_95%_)/D_average_.

For all patients the normal tissue was defined as an OAR by subtracting the PTV from the patient outline and used during optimization to prevent high dose areas outside the PTV.

Several planning studies have been published comparing fluence modulation with subsequent leaf sequencing IM and the direct aperture optimization DSS in Oncentra MasterPlan^®^, showing clear advantage for DSS [28-30]. Therefore, the reference IMRT plans were optimized with DSS in this study. Seven equispaced beams have been used in all IMRT plans.

For the optimization of VMAT plans, single arcs ranging from 182° to 178° gantry angle with a gantry angle spacing of 4° and the leaf motion constrained to 0.5 cm/° were used. The collimator angle was set to 45° as suggested in [34], except for the head and neck case for which the collimator had to be set to 0° to ensure PTV coverage. Maximum delivery time was set to 150 s per arc for patient number 1 and 2, and to 200 s per arc for patient number 3. If the plan quality achievable with single arc was not comparable to IMRT, plans were re-optimized using the dual arc option leaving gantry angle range and spacing unchanged. Dose volume objectives were kept identical to the IMRT plans. In addition to plan quality the times required for planning and irradiation were compared. Calculation times were measured from the start of the optimization until the end of the final dose calculation, irradiation times were measured from the start of the first beam until the end of the last beam.

## Results

**Figure 1:**
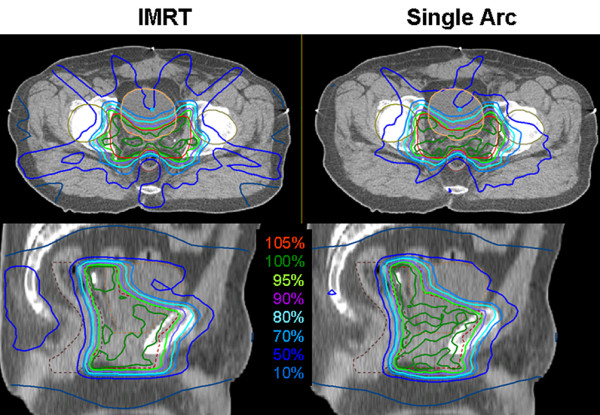
**Dose distributions for prostate cancer**. Comparison of dose distributions achieved with 7-field IMRT (left) and Single Arc VMAT (right) on representative transversal (top) and sagittal (bottom) CT slices. The PTV is drawn in red, the bladder in orange, the rectum in maroon, and the femoral heads in green. Isodose lines are shown in percent of the prescription dose, i.e. 60 Gy to the average of the PTV.

**Figure 2:**
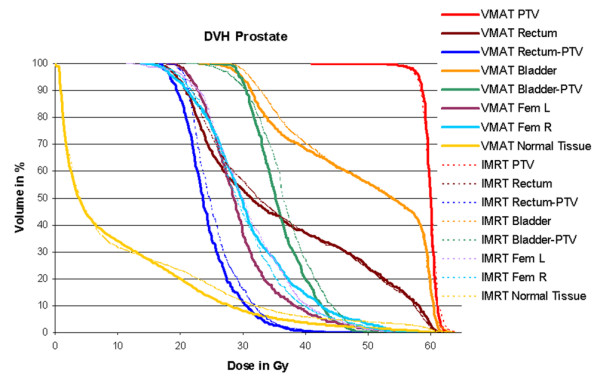
**Dose volume histograms for prostate cancer**. Comparison of dose volume histograms achieved with 7-field IMRT (dotted lines) and Single Arc VMAT (solid lines). Plan quality is slightly better for VMAT, with better target coverage and homogeneity and lower OAR doses.

**Figure 3:**
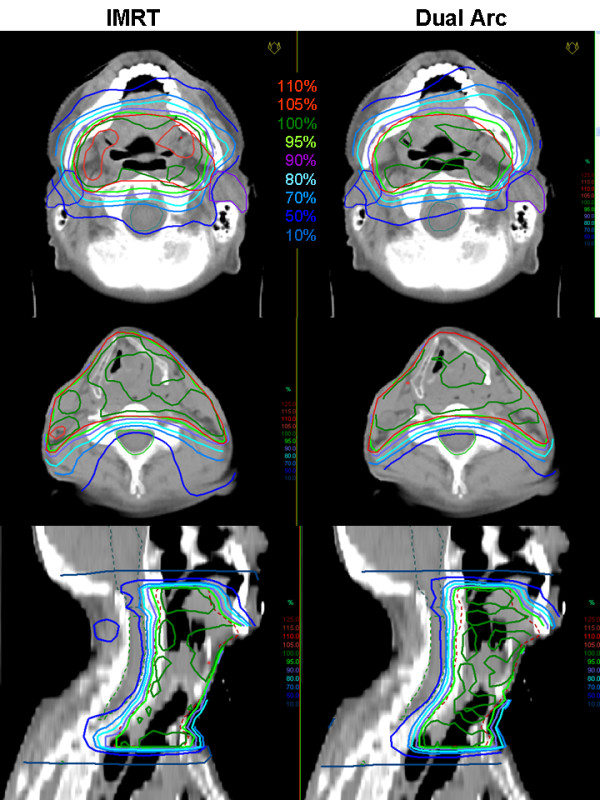
**Dose distributions for hypopharynx/larynx cancer**. Comparison of dose distributions achieved with 7-field IMRT (left) and Dual Arc VMAT (right) on representative transversal (top) and sagittal (bottom) CT slices. The PTV is drawn in red, the parotids in blue and purple, the spinal cord in green, and the brain stem in bright blue. Isodose lines are shown in percent of the prescription dose, i.e. 60 Gy to the average of the PTV.

**Figure 4:**
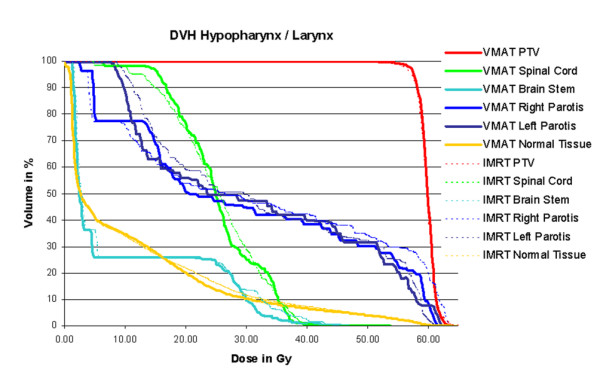
**Dose volume histograms for hypopharynx/larynx cancer**. Comparison of dose volume histograms achieved with 7-field IMRT (dotted lines) and Dual Arc VMAT (solid lines). Plan quality is slightly better for VMAT, with somewhat lower dose to the parotids.

**Figure 5:**
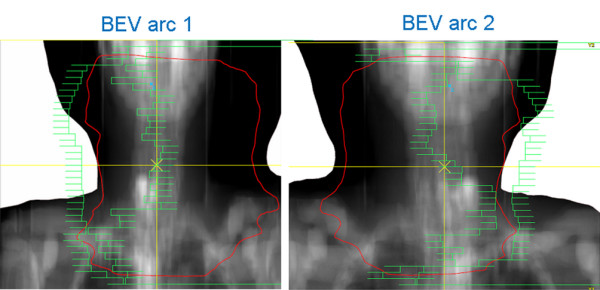
**Typical MLC positions resulting from Dual Arc optimization**. In Dual Arc VMAT, segments are grouped into arcs such that leaf travel is minimized during each rotation. In the example shown in this figure, arc 1 contains segments with leaves positioned more to the left, arc 2 to the right of the field.

**Figure 6:**
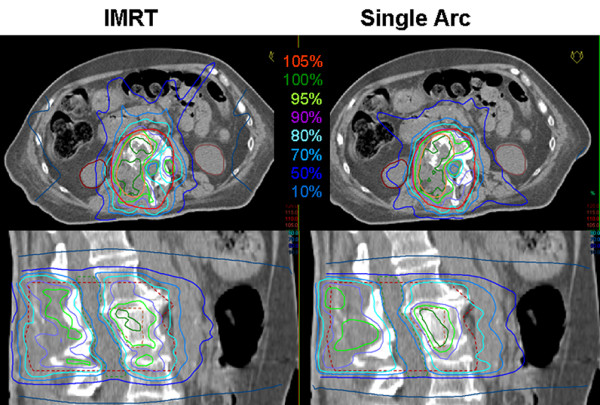
**Dose distributions for metastases in the lumbar vertebra**. Comparison of dose distributions achieved with 7-field IMRT (left) and Single Arc VMAT (right) on representative transversal (top) and sagittal (bottom) CT slices. The PTV is drawn in red, the GTV in orange, the spinal cord in green, and the kidneys in maroon. Isodose lines are shown in percent of the prescription dose, i.e. 55 Gy to the average of the GTV.

**Figure 7:**
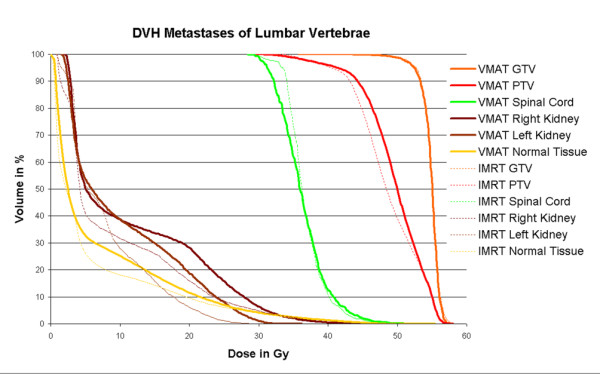
**Dose volume histograms for metastases in the lumbar vertebra**. Comparison of dose volume histograms achieved with 7-field IMRT (dotted lines) and Single Arc VMAT (solid lines). Using the same dose volume objectives for optimization, higher doses to the PTV are achieved with VMAT for almost identical GTV coverage and homogeneity and sparing of the spinal cord but somewhat higher dose to the kidneys.

The feasibility study showed similar plan quality at reduced delivery times and similar number of MU per fraction for VMAT as compared to IMRT in all cases:

1. For the prostate case, single arc VMAT showed better dose homogeneity and target coverage, and similar, mostly even lower dose to the organs at risk. Time for optimization and dose calculation increased by a factor of 5.8, treatment time was reduced to 43%. Detailed information is given in table 1. Figure 1 shows the dose distribution in transversal CT-slices, figure 2 the respective dose volume histograms (DVH).

2. For the case with cancer of the hypopharynx/larynx, single arc VMAT showed similar target coverage and better sparing of the parotids, but deteriorated homogeneity as compared to IMRT. Better overall plan quality including target coverage, homogeneity inside the PTV, as well as OAR sparing could be achieved with dual arc VMAT. Even the relative volume of the normal tissue, receiving doses between 20.0 Gy and 50.0 Gy is smaller in case of the dual arc treatment. Only the relative volume of the normal tissue receiving between 5.0 Gy and 20.0 Gy is slightly larger. Detailed information is given in table 2. Figure 3 shows the dose distribution for dual arc as compared to IMRT in transversal slices, figure 4 the respective DVH. Segment shapes for a selected gantry angle are shown in figure 5, illustrating the grouping of the segments into arcs with respect to the leaf positions. For dual arc, time for optimization and dose calculation increased by a factor of 6.8, treatment time was reduced to 35%, as compared to IMRT.

3. For the patient with metastases in the lumbar vertebra, single arc VMAT showed similar plan quality as compared to IMRT. Doses to the GTV were similar, median dose and D_95% _for the PTV higher, doses to the kidney were also higher but still below the tolerance and fulfilling the DVO used in optimization. Time for optimization and dose calculation increased by a factor of 5.0, treatment time was reduced to 41%. Since patients with bone metastases suffer from pain and are not able to keep the position for a long time, the VMAT plan was considered superior because of the reduced treatment time. Detailed information is given in table 3. Figure 6 shows the dose distribution in transversal, sagittal and coronal slices, figure 7 the respective DVH.

Patient 1 and 3 have actually been treated with VMAT after successful completion of commissioning, patient 2 had already finished treatment.

## Discussion

The VMAT optimization tool implemented in Oncentra MasterPlan^® ^v.3.3 allows creating VMAT plans with similar or better plan quality as compared to IMRT which can be delivered in substantially reduced treatment time on an Elekta SynergyS^® ^linear accelerator. For the treatment of prostate cancer and vertebral metastases, the required plan quality could be achieved with one single arc VMAT, which is in agreement with the results published for other types of equipment [4,18,24,35]. For the treatment of hypopharynx/larynx cancer, however, single arc VMAT did not lead to sufficient plan quality, reducing target homogeneity as compared to IMRT and violating the DVO for the spinal cord. The dual arc technique strongly improved plan quality as compared to single arc VMAT but also to IMRT, which also complies with publications to other VMAT solutions [14,24,36]. The findings of Bertelsen [25], who reported good results for single arc VMAT for head and neck cancer using SmartArc^® ^(Philips Healthcare, Andover, MA) could not be confirmed. In this case, however, plan comparison was performed for simultaneous treatment of three target levels, which requires certain dose heterogeneity inside the target.

The applicability of the system to simultaneous integrated boost concepts has been demonstrated for the treatment of vertebral metastases. In this case, a single arc was sufficient to achieve the required plan quality. The same concept can be applied for SIB treatments of other target types like prostate or head and neck cancers. It might even be possible that single arc treatments are in general suitable for SIB concepts due to the required dose heterogeneity inside the target, which would also explain the results of Bertelsen [25] mentioned above. This, however, remains to be investigated in a separate study.

In the VMAT solution implemented in Oncentra MasterPlan^® ^v3.3, segment shapes and weights are subject to optimization, which is one of the main differences to the treatment planning system ERGO++^®^: In ERGO++^® ^segment shapes have to be defined by the user prior to optimization, and only the segment weights are optimized.

The quality of the VMAT plans resulting from optimization in ERGO++^® ^is therefore highly dependent on the individual user's experience in creating suitable segment shapes. The VMAT solution implemented in Oncentra MasterPlan^® ^v3.3 in contrary does not require any user input for the segment shapes. Segment shapes and weights are resulting from the optimization process and the resulting plan quality is therefore less dependent on the individual user.

The number of monitor units per fraction in this study was similar for VMAT and IMRT, a significant reduction as reported for Varian linacs could not be observed [36], since the values found for IMRT were already considerably lower than the ones reported for Varian. Treatment times, however, could be substantially reduced to 35% to 43% as compared to IMRT, whereas calculation times were 5.0 to 6.8 times higher for VMAT.

The combination of plan quality and treatment time shows clear advantage of VMAT over IMRT: Treatment time is a crucial factor especially for patients who suffer from pain or are not able to keep a certain position for a longer time, as it is the case e.g. for patients with bone metastases, or for patients with significant internal organ movement, e.g. patients with prostate cancer, for which the actual delivered dose distribution might differ significantly from the planned dose distribution due to intra-fractional movement. In these cases even a single arc leads to the required plan quality, allowing reducing overall treatment time from 11 minutes to well below 5 minutes. For the patient with hypopharynx/larynx cancer the dual arc VMAT showed better plan quality at only 33% of the treatment time, which reduces patient discomfort in the rigid mask system. The reduction in delivery time leads to better patient comfort and possibly also quality of delivery, and simultaneously reduces the workload and increases availability of the linac.

The only drawback found for VMAT as compared to IMRT was the increased calculation time. This, however, has no impact on patient treatment or on the workload but is only affecting availability of the treatment planning station. Workload for the planner is virtually the same for VMAT as for IMRT, since the steps of the planning procedure, which require user interaction, like definition of structures, beam setup, definition of DVO, are the same in both cases. In the future calculation times may be reduced using a processor with more than 8 cores or performing the dose calculation on the GPU processor, as it will be implemented in the next version of Oncentra MasterPlan^®^.

It could be shown that VMAT planning with Oncentra MasterPlan^® ^has the potential to produce better plan quality requiring less delivery time as compared to IMRT. However, dedicated planning studies should be performed, varying the user definable parameters e.g. maximum treatment time, number of arcs, and gantry angle range, to identify the best parameter set to achieve optimal combination of plan quality and treatment time for each target type.

## Conclusion

Oncentra MasterPlan^® ^allows achieving comparable or superior plan quality with VMAT as compared to IMRT. Times required for optimization and dose calculation are increased, the number of monitor units per fraction is similar, and treatment times are strongly reduced.

## Abbreviations

CT: Computed Tomography; DSS: Direct Step and Shoot optimization; DVH: Dose Volume Histogram; DVO: Dose Volume Objective; GTV: Gross Tumour Volume; IM: Intensity Modulation with subsequent sequencing; IMRT: Intensity Modulated Radiation Therapy; MLC: Multi-Leaf Collimator; MU: Monitor Units; OAR: Organ at Risk; PTV: Planning Target Volume; SIB: Simultaneous Integrated Boost; TPS: Treatment Planning System; VMAT: Volumetric Modulated Radiation Therapy

## Competing interests

This work was partly supported by Theranostic.

## Authors' contributions

BD conceived of and designed the study, performed treatment planning and plan comparison and drafted the manuscript. KW performed part of the treatment planning. OK helped to draft the manuscript. All authors read and approved the final manuscript.
